# Selections

**Published:** 1875-07

**Authors:** 


### Selections.

(From the Psychological and Medico-Legal Journal.)

### THE NEUROTIC ORIGIN OF DISEASE, AND THE ACTION OF REMEDIES ON THE NERVOUS SYSTEM.

By FREDERIC D. LENTE, M.D.,

MEMBER OF THE COUNCIL OF THE NEW YORK NEUROLOGICAL SOCIETY.

Read before the New York Neurological Society, Dec. 7, 1874.

[Continued from page 460.]

*Quinine* has already been discussed in this paper in some of its relations, especially as regards its action on malarious and neuralgic affections. Its power of arresting or retarding the amœboid movements and migration of the white corpuscles of the blood, and thus preventing inflammation or suppuration, asserted by Binz, has been lately called in question by other observers, and his inferences, rather than his facts, discredited. He also claims, that, as quinine arrests putrefaction and fermentation by killing the spores or fungi which cause these changes, it will also arrest or relieve septicæmia. But his experiments on animals do not sustain this view. It is generally admitted, however, that large doses of quinine do have a remarkably beneficial influence on septic diseases ; and we can only explain it by its action on the nervous system—such an action as we have claimed for it in other affections. The antipyretic virtue of quinine, so highly commended of late, has also been denied, but principally, I think, in consequence of observations made on *healthy* men, which is a most fallacious guide, except where ocular inspection of the physiological processes can be had. Dr. H. Wood, who ably discusses this question of the various actions of quinine on the system, comes to the conclusion that "it exerts in febrile disease a decided but feeble antipyretic action." He, however, seems to draw his conclusions almost entirely from German sources. The experience of American physicians will be found more favorable. There is nothing, in all the various and ingenious experiments and observations, it will be noticed, which warrants the generally received opinion that it has any modifying influence on the blood itself, or any antidotal effect against supposed disease germs, either vegetable or animal. But on the blood-vessels its action is not so well settled. This is an important question, and has attracted the attention of our sister Society at a previous meeting, but the experiments and observations which had then been made* were too meagre to give much support to either side. The result of Dr. Hammond's experiments with his *cephalohæmometer*, was opposed to that of Dr. Janeway, who used ocular inspection of the vessels, through a similar opening in the skull. It is a little singular that, when Binz and others in Germany, were observing, in so many instances, the effect of quinine on the *contents* of the vessels, they should have made no note on the condition of the vessels themselves.

* If the effects of these drugs were produced through the medium of absorption into the blood, we could hardly reconcile such a theory with the above remarkable facts. It would, however, seem that a fluid preparation, like croton oil, lying for hours in the intestines, must be absorbed to a great extent ; but, supposing this to be so, it does not produce its characteristic effect, as we have seen by the result of injection into the veins.

But I am able now to supply to a certain extent this oversight.

I learned from Dr. George R. Cutter, one of the surgical staff of the New York Eye and Ear Infirmary, that he had occasion to examine into this matter in Germany, and he was kind enough to give me the results from memory, as he had lost his notes. I extract the following interesting account from his letter:

"While working in Prof. Julius Arnold's laboratory in Heidelberg, during the winter of 1870–71, a Russian, whose name I have forgotten, was making a study of the effect of quinine on the system. At his request I examined the dogs, rabbits and frogs with the ophthalmoscope, and made measurements of the retinal vessels, before giving quinine, as well as at various periods during the administration and afterwards. A gradual but marked diminution of the *arteries* took place, and before the death of the animals, the arteries became mere threads. The veins were also much diminished in diameter. The animals often became blind, and the retina was anæmic in the highest degree. The mesenteries of frogs were exposed under the influence of woorara and without. Quinine was injected into some of the frogs. The mesenteries were kept warm, and covered with serum. Under the microscope the effect of the quinine on the arteries was well marked. Inflammation was delayed. The *auswanderung* of the corpuscles was also very much retarded and diminished, and the inflammatory dilatation was lessened to a considerable degree."

These results were also *controlled* by Profs. O. Becker and J. Arnold, Jr.*

The effect on the corpuscles, mentioned by Dr. Cutter, agrees with the observation of Binz and others, to which I have just alluded.

*Strychnine* is one of the most important of the neurotics, since it stands as yet the sole representative of the motor-*excitants*, while we have a number of powerful motor-*depressants*. With regard to its effects on the

* Others have noticed this interference with the motion of the corpuscles, when experimenting with other agents which contract the capillaries, as digitalis and ergot; evidently due to the narrowing of their calibre, and finally, in the case of digitalis, to complete closure. I have said it is singular that Binz did not remark upon the contraction of the arterioles, if there was any, but he was looking for something else, namely, the direct effect of the quinine on the corpuscles, and of course attributed their tardy movement to this.

capillaries, it has been seen in one instance at least, according to German authority, to *contract* the vessels of the frog's foot. This cannot be accepted without further observations, since it is not what we would be led to expect from other experiments, and from clinical observations. That it induces hyperæmia of the cord is an accepted fact, and it is quite common to use a very small hypodermic injection as a diagnostic, when we are in doubt as to whether we have anæmia or hyperæmia. Clinical observation would also lead us to infer that it produces dilatation of the cerebral vessels, but I believe that no *experiments* have been made in this direction. I would here remark that probably some of the many discrepancies which are noticeable among experimenters of equal ability, with these active poisons, may be accounted for by the fact that the effect on the lower animals, of a *large* dose, which is generally intended, and an *overpowering* dose, or an *overwhelming one*, is often very different, and sometimes directly the reverse. This very drug affords an example, as the following quotation shows: "As Fraser has discovered for atropia, Vulpian has found for strychnia; namely, that after a time, say, from some hours to two days, if the dose has been of the right size, the strychnic *paralysis* passes off, the motor-nerves are found to have regained their power, and the convulsions reappear and continue for hours or days." H. Wood (op. cit.)

We have also seen, in the first part of this paper, that a certain degree or extent of impression on the surface of the human body may cause a *contraction* of the visceral vessels, while an impression, greater in degree or extent, may cause *dilatation* and rupture. That the influence of this drug is entirely exerted on the *nerve-tissue* of the spinal cord, in the production of the motor phenomena, and not through the blood, on the nerves themselves or the muscles, is well established by numerous experimenters.

Dr. S. Weir Mitchell informs me that he has lately performed the following experiment: Having bled an animal nearly to death, and also isolated a portion of the cord from its vascular connections, he applied strychnia to it, and its peculiar action was well pronounced, showing its action on the *nerve-cells*. We may, therefore, deduce this valuable therapeutical inference, that in cases of spinal or cerebral anæmia with impairment of nutrition of nerve matter, we may employ this remedy for a double purpose, to act *directly*, and also indirectly by increasing the supply of blood. The prompt and direct conveyance of the strychnic influence from the surface, through the afferent nerves to the spinal cord, and thence to the most distant parts, is proved by the often-repeated experiment of isolating the sciatic nerve in the frog, by a section of all other tissues, and then applying the poison to the foot. It is promptly conveyed to the spinal cord, and its effects manifested in the other limb and elsewhere. I refer to the two following clinical observations as illustrations of the value of the application of the results of this experiment to actual practice.

The treatment of the following case I had the opportunity of watching through the courtesy of Prof. Hammond. The history I had from the patient himself.

CASE I.—Mr. W., a grain inspector of Chicago, was attacked three years ago with epileptic convulsions; has had them once a month or oftener; also some threatening cerebral symptoms; had no treatment that he knows of except moderate doses of bromide of potassa and chloroform inhalation. In June last he had a recurrence of cerebral symptoms, insomnia, pain, double vision, etc. This lasted two weeks and disappeared. On the 14th July, after some exposure to the sun, he was again attacked with the above symptoms, to a greater degree, and with complete inability to raise the eyeball or upper eyelid (left eye), also extreme internal strabismus, diplopia and severe cephalalgia. These symptoms occurred suddenly in the night. Could neither read, nor distinguish the quality of grain. The strabismus disappeared slowly, and the ptosis also diminished somewhat, so that when he applied to Dr. H. about the 13th October, 1874, he could, *by an effort*, raise the lid so as to expose the cornea, but it fell back immediately; other symptoms the same. He was put upon increasing doses of the iodide of potassium, with the idea of relieving the *basilar meningitis*, presumed to be the cause of the symptoms, the application of the induced current to the brow and temple, and the hypodermic injection of strychnia. No *immediate* effect could be expected from the first two remedies; it is to the last that I desire to direct attention. Prof. Hammond proposed to inject the solution directly into the affected muscles, and accordingly did so, using grs. $\frac{1}{32}$ in two drops of water; it is presumed that it passed into the muscle, or most likely in its immediate proximity. In all, six injections, I think, were used. I

watched the effect carefully, and tested the eye and lid after each. They were done each alternate day. He declared that he perceived quite a decided effect. After the second there was no doubt, as I could see the change within fifteen minutes both on the ball and on the lid, but especially on the latter; after the third, the *ptosis* had entirely disappeared, and he could raise the *ball* to a horizontal plane; the diplopia had disappeared, and he could read by holding the book low. After the fifth injection (gr. $\frac{1}{20}$) no difference in the appearance of the eyes was distinguishable, and he could read with the book held directly before him. He considered himself cured.

A case of almost perfect deafness, incapacitating the patient entirely from business as a lawyer, partly from Eustachian trouble and partly from an affection, apparently, of the auditory nerves, was treated by Dr. Hammond under my observation with a striking effect. I could converse with him, after two or three injections, at a distance of two feet, with the voice slightly raised, and speaking very distinctly, with no difficulty; but, in cases of deafness, it is so easy for a patient, who is anxious to improve, to deceive himself and others, that I should wish to see or hear from him a month afterwards, before reporting a cure. He, however, returned to his home in Buffalo, with his brother, both convinced of very marked improvement, if not cure.

CASE II.—(From the private note-book of Dr. C. R. Agnew—notes abridged.

*Amblyopia, from tobacco and alcohol.*—On testing vision, it was found to be $\frac{14}{100}$ with either eye, and not improved by glasses. Both visual fields perfect, and no color scotoma. On ophthalmoscopic examination, both eyes emmetropic, and in each eye that dirty, ill-defined appearance of the nerve and retina, which, taken in connection with diminution of the vision, without other evident cause, usually leads us to suspect alcohol and tobacco poisoning. Directed him to abstain entirely from alcohol and tobacco, and to return in a week. Oct. 30, one week later, R. E., V. $\frac{20}{100}$, L. E., V. $\frac{20}{80}$. Injected strych. int. gr. $\frac{1}{36}$, and in one minute vision rose to $\frac{20}{50}$ with both eyes; and five minutes later to $\frac{20}{40}$. The injection was in the cellular tissue, but not in that of the affected part. In Dr. Hammond's ear case, it was in the arm. I do not suppose that the medulla spinalis was affected through the rapid absorption into the blood, but that the effect was transmitted to it, as electricity would be, and as, in the case of the sciatic nerve of the frog, almost instantaneously. Nor do I suppose, even if the solution reached Mr. W.'s superior rectus muscle, which it probably did not, that it affected the muscle *directly;* for it is now an unquestioned fact, that if the leg of a frog is amputated, and a solution of strychnia injected into the artery, no effect on the muscles follows. It must take place, if at all, through the spinal cord, and cannot reach it through the blood, as has been demonstrated by the late experiment of Weir Mitchell, and by others.*

The effect of *atropia* on the vaso-motor system, and probably on the nerve-tissue itself, is scarcely less interesting and important than that of ergot and strychnine, but I am compelled to abstain from the more thorough analysis of its physiological and therapeutical action, as well as that of the other valuable drugs enumerated in the list, in an early part of this paper which was proposed ; in consequence of its unexpected length, which was wholly unintentional, but could scarcely have been avoided. For the same reason it will be advisable to defer reading the *cases* appended to this paper, and which were relied upon, in some measure, to corroborate the facts and opinions here and there given, without a due amount of evidence. With ergot, atropia has been lately relied upon as our best resource for reducing the calibre of the capillaries, and thus relieving inflammation.

* As illustrative of the action of strychnine in these cases, I refer to a case published by me in the *Am. Jour. of Med. Sciences*, 1862, of injury to the branches of the supra-orbital nerve, by a fragment of copper cap, causing partial amaurosis, and immediately relieved by removing the foreign body; also other cases of amaurosis caused by trivial cicatrices of the scalp, and relieved by excision. Handfield Jones states cases of paralysis of the muscles of the eye from neuralgia. (*Practitioner*, March, 1874.)

Mr. Charles Hunter, of London, who has written largely on hypodermic injections, makes a statement calculated to injuriously circumscribe the applicability of strychnine in these cases, to the effect that if improvement does not follow three or four injections, the case might as well be regarded as hopeless, so far at least as this remedy is concerned. This statement is contradicted by my own experience, by that of Prof. Hammond, especially in cases of infantile paralysis, and by several American oculists. In fact, the proper dose cannot be ascertained until several have been given, as the toleration of different patients differs materially.

The effect of the little gelatine wafers, used by oculists to dilate the pupil, is similar to that claimed for strychnine, otherwise it seems incredible how the 1-20000 part of a grain of atropia should act, as it does, almost immediately. If it is claimed that it acts through the blood, then the disciples of Hahnemann might well claim this as a proof of the efficacy of any of his attenuations.

The physiological experiments on animals are very numerous, and the number of distinguished experimenters large. An excellent *resume* of these interesting experiments will be found in H. Wood's work, so often referred to, which conclusively demonstrates the fact of a decided contraction of the arterioles, not from the direct effect observed under the stimulus of atropia on the frog's foot, or the mesentery, but mainly from the uniform increase of *blood-pressure*, from contraction of the capillaries, and the absence of this after section of the medulla. Dr. Wood has also repeated these experiments. —*Am. Jour of Med. Sciences*, April, 1873. One of the clinical histories, which were to have been appended to this paper, affords a striking exemplification of this fact. The excessive congestion of the lungs, and extravasations therein, and even of the retina itself, shown in post-mortem examinations of those dead from immense doses of atropia, indicate, as has been indicated in the case of other alkaloids, the importance, in estimating their physiological effects and therapeutic value, of discriminating between the phenomena produced by moderate and by inordinate doses respectively. Atropia has lately been highly recommended in excessive sweating, especially of phthisis; we all appreciate how annoying and intractable a symptom it is, especially since we cannot usually remove the cause. Supposing it to be due to a vaso-motor paralysis of the *plexus* supplying the sudoriparous glands, we should expect a remedy like this to act promptly on its nerves, diminishing the lumen of the vessels, and thus arresting the hypersecretion more certainly than the ordinary astringent remedies usually given, and which act through the uncertain medium of the blood, in which they must necessarily be largely dilated. Besides this, our most reliable remedies are very *hot* water to the surface, producing a reflex action, and the old-fashioned sage-tea. Had we the active principle of the sage, or volatile oil, so as to employ it in more definite and reliable doses, it might succeed even better. The great value of atropia in opium-poisoning is now so generally admitted, though still denied by many writers, that it is hardly necessary to allude to it. I have published several striking instances myself, and would refer any skeptics to Dr. Trask's account of his own case (*N. Y. Med. Journal*, Nov., 1874). He took a dose, by mistake, estimated at six to seven grains; and I heard him read an excellent paper recently before the

Obstetrical Society of this city, indicating no damage to body or mind. Electricity and atropia saved his valuable life.*

The consideration of *opium* naturally succeeds that of belladonna. M. See has lately placed morphia with strychnine as a "motor of the spinal cord." Recent extensive experiments in Germany go to show that the local application of morphia increases and protracts the excitability of the nerves induced by strychnia; and its action on the brain, in certain doses, not too large, is similar to that of strychnine. Opium has not yet been proved experimentally to dilate the capillaries, but clinical observations lead us to infer that it does. We have seen how prompt is the action of belladonna in the relief of sweating, and we know how certain the action of opium is in its production. The idea that opium does produce hyperæmia of the brain has had a firm hold on the minds of the profession from the earliest period, and has had a very unfortunate influence on the treatment of cerebral affections, especially in childhood, and on a large number, perhaps, whose convulsive phenomena are due to mere *irritation* of the meninges from a morbid influence applied to the peripheral nerves in a distant part of the body, generally in the abdomen. Though opium may physiologically dilate the cerebral vessels, and though a positive inflammation of the encephalon may exist, if the pain is excessive, the restlessness and insomnia protracted, and uncontrollable by other means, as cold, bromides, atropia, etc., as is too commonly the case in acute forms, opium is our only resource; it may have to act in some desperate cases, by its overwhelming effect. But it may generally act in a legitimate and rational manner. The old maxim, *ubi irritatio ibi fluxus*, will explain it. The very pain and its consequences cause or increase the flow of blood, and the relief of pain and the allaying of excitement restore, to a considerable extent, the balance of the circulation. The truth of the above maxim is well illustrated in facial neuralgia, which cannot be charged with being inflam-

* Two remarkable cures by belladonna of exophthalmic goitre, in which the symptoms had assumed such proportions as to threaten death, are reported in the *Lancet*, 1874, by Dr. Smith, of London. Various other remedies had been used in vain. Profuse sweating in both was a prominent symptom. Five drops of the tincture were given hourly, and there was a rapid and progressive fall in the frequency of the pulse. At the end of ten days a drachm was given daily.

matory; when excessive, the face becomes red and swollen, looking as if it had been struck; a full and effective anodyne relieves the hyperæmia as well as the pain. No matter what the disease, whether attended by fullness or anæmia of the vessels, if pain or spasm is uncontrollable by other means, opium is a proper remedy. Even in the profound coma of *uræmia*, opium, by allaying the irritation of the brain, produced by the poison, will rouse the patient out of it. In the convulsions and coma attending or following the puerperal state it is our best remedy; though, when the paient is in good health, the old plan of bleeding, no doubt, aids materially by removing a portion of the poison with the blood. It is hardly necessary to say that, in all these cases, the action of opium is on the nervous system, and has no influence whatever in the blood, which is its vehicle merely.

I have included the *anæsthetics* in the list of neurotics, because their action, however we explain its rationale, is on the nerve-centres; it is through them that they produce all their marvellous effects, and it is to them that we must look for explanation of the fatality which attends the use of some of these agents, and to which we must direct our means of resuscitation when death has occurred, or is imminent. On the capillaries, both ether and chloroform have been proved, by experiments and observations, to act like atropia, ergot, etc., to contract them; we need not fear to give them, therefore, in congestion or inflammatory conditions, though the struggles, which are apt to precede full anæsthesia, may temporarily increase the fullness of the cerebral vessels. Various explanations of the cause of anæsthesia, and of death from it, have been given, but none of them are thoroughly satisfactory. In the few cases of death from ether, it has been noticed that respiration is first arrested, and some time after, the action of the heart. The reverse is the case with chloroform; with ether, the effect is slow, and preceded by symptoms, which, with watchfulness, might enable us, perhaps always, to ward off a fatal result; with chloroform, it comes like a stroke of lightning, the heart is paralyzed in an instant. How can so sudden an action be explained? Only, I think, by a reference to a reflex influence on the nerve-centres. Because the application of pure chloroform to the naked heart, or the injection of pure chloroform into the jugular vein, has produced instant paralysis, it has been assumed that the paralysis in inhalation is also caused by direct action through the blood; it is surprising that one who reasons so well, and analyzes so well as Horatio Wood, should adopt this explanation. The charging of the blood by any anæsthetic must, under any circumstances, be a slow process, and a sudden effect could hardly be expected; and the paralysis, produced by the direct application of so irritating an agent as chloroform, whether to the surface of the heart, or internally, through the jugular vein, is more likely caused by reflex action, which would just as soon follow ammonia or nitric acid; just as when atropia was applied to the frog's foot and contraction of the capillaries followed, it was assumed that it was from its direct action on the vessels, but subsequent experiments, with the skin above the foot divided, showed that it was from reflex action. On what peripheral nerves, then, does the chloroform act to produce so powerful a reflex influence on the nerve-centres and thence on the heart? I answer, on those of the air-cells and the bronchioli; and the reason why it does not *always* produce this effect is, that it is generally largely diluted with air; and, a greater protection still, because the air, entering the lung at each inspiration, bears but a small proportion to that already in the lung, the *residual air*, and this therefore can be but slowly charged. But this care in diluting the anæsthetic with air, notwithstanding the evidence which is so satisfactory to a coroner's jury, is, as we all know very well, sometimes forgotten in the interest of the operation, and, now and then, I fear, in sheer recklessness, and the full force of the vapor, by an unusually forcible inspiration, is carried deeply into the lung, and, acting on nerves already pretty thoroughly impressed, induces the sudden reflex phenomenon which we frequently see exemplified by other accidents on other parts of the system. Walshe says: "Saturation of the nerve-centres is the essential and radical cause of the event." This is high authority, but he does not advance any proof, and the effect is too sudden to give the idea of saturation, through the blood. Whatever the impression may be, it must be something sudden and powerful, generally acting on tissues already tolerably saturated, but not necessarily so, as the effect is sometimes produced at the commencement of the inhalation. Paralysis of the heart is an exceedingly common accident from a great variety of causes, mental and physical, and occurs as the cause of death, and often as the unexplained cause of death, in many diseases. It occurs in animals as well as in men, from mental emotion alone; in the former, from fright, of which there are numerous examples; in the latter, from pleasurable as well as painful emotions. Of the former, a little bit of revolutionary history will serve as an example. When the surrender of Lord Cornwallis was announced suddenly to the door-keeper of Congress, he fell dead. Similar examples are on record. Fright, we all know, will suddenly, by acting on the nerve-centres, relax the sphincters, and act on the bowels, and also produce sudden death. A blow on the epigastrium, transmitted to the semi-lunar ganglion, or the crushing of an extremity, (Walshe), will paralyze the heart.* The effects of the inhalation of ether and chloroform respectively, and likewise of the slow and sudden inhalation of chloroform, may be likened to those of the gradual and the sudden annihilation of the brain, as detailed in the experiments of Legallois, in which he showed that the heart continued to beat after he had removed the entire brain, piece by piece, *slowly*. But, that instant paralysis followed *sudden* crushing of the encephalon. This may, I think, be considered an *experimentum crucis* in sustaining the theory advanced in this paper as against that of the direct action on the heart (Wood) or the gradual saturation of the nerve-centres (Walshe).† Flint says that

* As additional evidence, for those who require it, of the important and immediate influence we may exert, by our remedial applications, whether by heat, cold, mechanical violence, as by cupping, electric shocks, etc., or by drugs, I refer to p. 437 of Flint's Physiology of the Nervous System. "Brown-Sequard and Lombard found that pinching of the skin on one side was attended by a diminution of the temperature in the corresponding member of the opposite side; and that, sometimes, when the irritation was applied to the upper extremity, changes were produced in the lower extremity. Tholozan and Brown-Sequard found also that lowering the temperature of one hand produced a corresponding depression in the heat of the opposite hand, without any notable diminution of the temperature of the whole body. Brown-Sequard showed that, by immersing one foot in water at 41° F., the temperature of the other foot was diminished about 7° F., in the course of eight minutes." These facts show that certain impressions made upon the sensory nerves affect the animal heat by reflex action. Also that the application need not be made in the immediate vicinity of the part designed to be influenced.

† B. W. Richardson says death is produced by the action of the vapor on the peripheral nervous system. But he explains it in this way. The inhalation suspends respiration "for an interval, then accumulation of carbolic acid in the blood, then irritation of the vagus, then arrest—from the irritation—of the action of the heart."

Why not irritation of the vagus *primarily* (by the chloroform) rather than *secondarily* (by the carbonic acid), since the former is certainly more irritating than the latter? And the sudden character of the death would rather sudden or powerful galvanization of the *pneumogastrics* will so excite their inhibitory action as to endanger heart paralysis. "The effects of pressure on those nerves in the human subject," says Flint, "is described by Aristotle, quoted by Waller. In some cases the patient falls instantly, as if struck by lightning, while in others the effects are not so marked." Waller performed several painless operations under this mechanical anæsthesia.

Dr. Marion Sims, in his paper recently read before the British Medical Association, attributes the danger to *cerebral anæmia*, and highly extols Nelaton's plan—suspension with the head downwards, by which a patient of his was restored to life. Of all single means of resuscitation this is probably the best. But we must go behind the anæmia to find out the source of danger; for this is due, as has been suggested, to cardiac paralysis, and behind this is the fatal chloroform vapor acting through the peripheral nerves (of the pneumogastrics); so on the nerve-centres, especially the *medulla oblongata*, exciting their inhibitory function, through the *par vagum*, on the cardiac ganglia and plexus. So that while we invert the body, we should also endeavor to rouse the heart's action by electricity applied to the pneumogastrics; and by applying the electrodes just above the clavicles, on either side of the neck, we may act upon the phrenics at the same time, thus exciting the diaphragm, and inducing respiratory efforts. This, in all probability, would have saved Dr. Sims and his illustrious confrere a world of dreadful suspense. With these measures might also be combined the injection of stimulants *per rectum*, or *intra-venous* or even of ammonia, after the manner of Halford, the use of digitalis, and of nitrite of amyl.

Cardiac paralysis, or a tendency to it, *cardiac asthenia*, as Fothergill* aptly terms it, is so common and so indicate a direct than an indirect process. It ought not to be forgotten, in estimating the effects of chloroform, that it *directly* tends to produce cerebral anæmia, by contracting the vessels; as does ether also. It is probable that force and fullness of the heart's pulsation, and diminished frequency, so commonly consequent on full anæsthesia, is due to a general contraction of the arterioles or the capillaries, and a consequent rise in arterial tension, and that the remarkable feebleness of the heart's action, which not uncommonly follows recovery from anæsthesia, is due to the sudden expansion of those minute vessels, and rapid drain on the encephalon. Here the nitrite of amyl would seem to be *the* remedy. Max. Schueller's recent experiments on animals demonstrate that this drug can remove the effects of chloroform on careful vascularity very promptly.

* "Digitalis: Its Mode of Action and its Use." By J. Milner Fothergill, London.

dangerous a complication of a great variety of diseases, that it demands further notice. Many times have physicians been astonished and shocked at a sudden and inexplicable "turn" for the worse, or a sudden death, in a case of acute disease, when all seemed to be going well; and their astonishment has not abated when, on a post-mortem inspection, they have been able to discover no sufficient cause of death. Two cases have, within the past two weeks, occurred in my own practice, which may serve as examples:—

CASE I.—L. T., a stout, healthy, temperate young man, had single *pneumonia*, and was attended, in my absence, by Dr. J. C. Young, late Resident Physician of Bellevue Hospital. The case progressed as usual, and in the course of a few days resolution commenced; then the other lung was invaded, but he showed no unusual or alarming symptoms; was taking stimulants very moderately. At 9 o'clock P. M. was seen by the doctor; pulse, 104; fair strength; respiration not particularly bad. At 12 P. M. he became weak and restless, then delirious. The doctor was called at 4 A. M., and found him with a feeble, frequent and irregular pulse, and he died in almost twenty minutes, although he swallowed the stimulants freely given.

CASE II.—Notes furnished by Dr. Murdock, who treated the case.

October 23. Mrs. M. C.—seventh day of single *pneumonia*. Doing well, and resolution commencing. Had taken nourishment abundantly, and had not required any stimulants. To-day found her pulse had risen from 100 to 116, and was somewhat feebler. Ordered brandy, ℥ iij, daily. Called in haste to her at 9.30 P. M. Found that she had steadily grown worse through the day, and for some hours alarmingly so. I found her almost pulseless, the heart's action too feeble, rapid, and irregular to number. Surface bathed in cold sweat, extremities cold, inspirations frequent and shallow, mind wandering. Ordered heat to extremities, and warm blankets. I gave three drachms of brandy every ten minutes. This was kept up at intervals of fifteen to twenty minutes, for nine hours. She also had a half ounce in beef tea, *per rectum*, every hour and a half, and *ammon. carb.*, gr. x, 2 h. Her case seemed utterly hopeless for some hours. It was not until near midnight that the skin became at all warm, and the pulse sufficiently strong to be numbered, still irregular. At 1.30 A. M. pulse

120, and more regular; skin warm. October 24, 9 A. M.—Stimulants have been continued steadily in same doses. Pulse 120, but quick and feeble. Three P. M., patient becoming hot and flushed. The brandy was given only every half hour, and the enemas were not retained, and had to be discontinued. Now, pulse feebler, and constantly intermitting. Continued brandy every twenty minutes, carbonate of ammonia, and also *tinct. digitalis*, m. x, q. 2 h. Six P. M., improving, pulse more regular; to give brandy every thirty minutes and digitalis every hour and a half. October 25, 8 A. M.—Much improved. Pulse 108, and quite regular. Brandy as before. Digitalis q. 2 h., and stop ammonia. Eight P. M., improving; digitalis q. 3 h. October 26, pulse 98; stop digitalis. From this date she continued to improve regularly. Attention is called particularly to the fact that there was no apparent change in the condition of the lungs to account for the sudden failure of the heart, and that, with the constant administration of stimulants, by mouth and rectum, for twenty-four hours, no marked improvement resulted until the digitalis was commenced, and almost immediately a favorable change was inaugurated.

Dr. Young calls to mind eight cases of pneumonia, two noted as double; and his successor remembers two others, and a case of Bright's disease, during his term of service (two years), in which sudden death occurred while the patients were walking to the water-closet, or merely getting on their feet, and of course not considered in a particularly dangerous condition. A case is related in the August number of the *New York Medical Journal*, as having occurred at the Charity Hospital, in which sixty-four ounces of serum had been drawn from the pleural cavity by aspiration, and eight hours after, the patient having been in the meantime comfortable, and sitting up part of the time, fell dead while walking to the water-closet. Prof. Hammond lost two cases, while in the army, in this manner; and a lady, under his treatment and that of Dr. Sims, for the opium habit: she died while waking across the floor. In convalescence from diphtheria, and during the attack, though the patient may not seem particularly weak, and able to be about the room, sudden death from this accident is very common. The first child I ever saw with it died during convalescence, from *taking her up*, contrary to express order, to pass water; and another, whom I had seen a few hours before walking about the floor, but with patches of dense membrane covering the pharynx, fell dead while walking from the sofa, on which I had directed he should be kept in the recumbent posture, to where his father sat, a few feet away. A lady, upon whom I had performed artificial delivery for convulsions, three days before, who was progressing favorably, and who was expressly ordered not to raise her head, feeling pretty well, sat up to take some drink, and fell back gasping, and was dead in an instant. Dr. North, then of Bellevue Hospital, who was assisting me in my practice at the time, was promptly by her side, but she could not be revived. She was excessively pale. These cases are all recent, and might be multiplied by consulting the journals and records of hospitals. Of course, some will attribute this accident to heart-clot. The autopsies in the hospital cases only showed clot in one. In diphtheria, we know that paralyses, in all parts of the body, are common. And the appearance of the patient's countenance in the other cases indicated paralysis rather than clot. Besides, when we do find clot, it is probably, in most cases, *post-mortem*, or forms in the death struggle.

"In this condition," says Fothergill (op. cit.), "we find the pulse furnishing us with the precursory evidences of approaching death." A very frequent, a weak, and an irregular beat. Clinical evidence informs us that this is most apt to occur in thoracic diseases, especially pneumonia. Why it is so is very evident, and it is hardly necessary to repeat the very lucid explanation of Fothergill. Suffice it to say, the lungs imperfectly aerate the blood, which flows *slowly* into the left ventricle, while the right becomes distended. "We have the blood more than ordinarily laden with carbonic acid, an agent which has a direct effect in paralyzing the heart, when brought in contact with the endocardium." An experiment of Cyon will illustrate this. He passed a stream of serum charged, and then one not charged with carbonic acid, and thus paralyzed and then restored the contractions at will.* There are all grades of this *cardiac asthenia*, from impending paralysis to a point where the want of correspondence between the state of the pulse and other symptoms is barely noticeable; and between these two degrees will be found a large proportion of cases, in which a remedy directed expressly to this peculiar heart-trouble may strike the balance between life and death, or may greatly

* Sydenham Soc. Year-Book, 1867–68.

curtail the duration of convalescence. It is in these cases that stimulants act so well, so greatly insisted on by Hughes Bennett. But frequently stimulants are too slow; and here digitalis, even by hypodermic injection, if necessary, proves its great superiority. "The question of special stimulants to the cardiac ganglia has," says Fothergill, "scarcely ever been broached." In these desperate cases, we must remember that *opium* also may afford material aid, and that it seldom acts, unless in unreasonable doses, otherwise than favorably on the heart; and if jactitation or pain be present, a hypodermic injection should be used.

I had a most remarkable exemplification of the value of opium in advanced heart-disease, a few years since. An old man, known to everybody in the neighborhood of Cold Spring as Johnny Moon, had severe valvular disease of the heart for nine years. Finally dropsical symptoms set in, and he took to his bed, and after having exhausted all the ordinary remedies, with temporary relief, finding his sufferings very great from the great amount of effusion and debility, I gave his wife a solution of morphia, to be used *pro re nata*, and no other remedy. I did not happen to see her for several weeks, when I met her, and to my inquiry she said, "Oh! doctor, that last medicine is curing him." And, surely enough, the effusions everywhere had entirely disappeared, and for some years after he was apparently quite well. But the heart-sounds were, of course, unchanged. I was in the habit of exhibiting him to my medical friends as a curiosity. He did not, after all, die of heart-disease or dropsy.

The cases of delirium tremens to which digitalis is applicable, and for which doses of two and four drachms even have been prescribed successfully, are those of cardiac asthenia, and indicate the boldness with which it must sometimes be given. Here the hypodermic injection would probably be safer. In very dangerous cases of suppression of urine, when digitalis, in the usual doses, fails to act, I have used poultices of digitalis, made by wetting flannels in a very strong infusion—four ounces to a quart—and using all the leaves (and none but the best English should be used) in one poultice, which extends all around the body, and from the thorax to the pelvis. H. Wood cautions against the danger of making the application too extensive and too strong; but I have used it as above, both in children and adults, and never saw any ill effect. However, no one should employ any of these powerful agents, now under discussion, without bearing in mind that he is dealing with a two-edged and a very keen-edged weapon; and that, when we speak of giving these fearlessly, and in such bold doses, it is generally in cases of imminent danger, where delay or pattering treatment is the most hazardous of all.

The *nitrite of amyl*, a remedy of recent date and not yet officinal, has already been alluded to in connection with the effects of other drugs. It is a most valuable addition to our neurotics, since its action on the nerve-centres is prompt, uniform and decided, and, with moderate care, it is quite safe; patients afflicted with epilepsy or *angina pectoris* being constantly trusted to use a regulated dose themselves by inhalation. In all spasmodic conditions it is invaluable. Prof. Janeway recently related the particulars of a case occurring at Bellevue Hospital, of a patient "moribund from advanced Bright's disease. He was unconscious, pulseless, pupils widely dilated, no sensitiveness of conjunctiva; twenty-five drops restored consciousness. The pulse gradually became full, and the patient could talk," and seemed like one restored from death to life. This is precisely similar to the effects produced by the transfusion of moribund cholera patients with saline solutions. The truce is but a brief one, however. Its importance as an auxiliary in the cerebral anæmia of chloroform poisoning, and of other similar and not necessarily fatal conditions, has already been noticed. As strychnine is used to diagnose doubtful conditions of the circulation in the *cord*, so we may use this drug for the *brain*, and Weir Mitchell* has used it for this purpose; Prof. Hammond also. Quite recently I have seen two cases in the practice of the latter, of epilepsy, in which the drug evidently precipitated the convulsion, and it was necessary to abandon it. Crichton Browne, in his large experience with this remedy, has, I believe, met with no such cases. This drug produces three most marked physiological actions, and with remarkable celerity and uniformity; first, dilatation of the arterioles, consequent sinking of blood-pressure, and of the action of the heart; secondly, loss of functional power, even to complete abolition, of all muscular tissues; thirdly, remarkable fall of temperature. Dr. H. Wood (op. cit., p. 299) says: "I have seen a pigeon per-

* Medical and Surgical Reporter, July, 1874, p. 69.

fectly conscious, although his temperature had been brought down by this agent some 13° F." As he states, that this effect is equally marked in fever, it may prove valuable as an antipyretic. This remarkable fact seems, at first glance, to be in direct conflict with the theory supported in this paper, that the rise of temperature in fevers and other diseases is due, in a great measure, to a dilatation of the smaller vessels, arterioles and capillaries, to a more rapid consequent metamorphosis of tissues, and that the action of our antipyretic remedies is mainly through their constringing effect on these vessels. But a *fourth* very remarkable property which this singular remedy has been proved conclusively to possess, harmonizes this apparent antagonism. It had been observed that in animals poisoned by it, the color of the arterial and venous blood was almost the same. Dr. Gamgee, by a series of beautiful experiments, has shown this to be due to the power of nitrite of amyl, in almost completely annihilating the oxidation of the blood corpuscles, and, to that extent, interfering with tissue change and the production of animal heat. A few drops of amyl diffused through a jar containing glowing phosphorus will extinguish it at once (Wood).

Three, four or five drops, by inhalation, from a piece of lint or a morphine bottle, constitute a dose for the most violent diseases. A case of puerperal eclampsia was arrested, as the *convulsions* would come on, by a single drop. But the secondary effects must be looked to here, as in chloroform or other inhalations under similar circumstances, namely, the danger of post-partum hæmorrhage. To *tetanus*, in connection with the physostigma or conia, and the bromides, it would seem to be particularly applicable, and some experiments on animals confirm this.

The action of *iodine* on some nervous diseases, and especially on the nervous manifestations of syphilis, is so remarkable and important as to justify considerable attention, if this paper had not already assumed unwieldy proportions. I will merely ask your attention to the fact that, though the iodide of potassium is a specific in these affections, and the bromide for certain other nervous maladies, neither the iodine nor the bromine, nor the potash alone, will have any decided influence whatever. Nor do we, as a general rule, see a *gradual* amelioration of the symptoms, but, after giving one, two, three, four, five, and sometimes six or more drachms of the *iodide*

per day, in gradually increasing doses, we see a sudden and very marked beneficial effect. And yet, if, while this tentative process is going on, we test the urine and other excretions, we find our remedy passing out of the system almost as fast as it is passing in, and, therefore, apparently not producing its effect by cumulative action. In the present state of our knowledge of the subject, there would seem to be but this explanation—that the iodine, when given alone, becomes rapidly acted on by the various chemical agents with which it comes in contact before reaching the nervous system, but, that, in the form of iodine, it is *protected* from change until it reaches its ultimate destination, where, under the influence of changes going on in the process of tissue-formation, the salt is decomposed, and iodide set free. It has been shown that medicines in a *nascent* state have a peculiar effect. Could we, in any particular case, know the exact dose without trial, very few of these efficient doses would doubtless effect a cure; since it is evidently not the blood on which we act, and, moreover, during the time in which we are finding our dose, we are interfering with the nutrition of the tissues. I may also instance the effect of large doses of the iodide of iron in some obstinate forms of hæmorrhage, especially uterine, and in nocturnal enuresis of children. It is not always that iodine requires to be administered in these very large doses to produce its decided effects on the nervous system. For instance, in spasmodic asthma, I have repeatedly relieved the most violent attacks, after failing with various remedies, by a dose or two of four or five grains, and within an hour or two. The noted Jonas Whitcomb's remedy probably owes all its success to this drug.

The *bromides* have already been incidentally alluded to. There is one rather widespread error, however, regarding their action, which tends greatly to circumscribe their usefulness, namely, that in cases of cerebral anæmia, or a condition of the circulation in which it may be supposed that this exists, they are useless and even injurious. For instance, in an abstract of a paper on the bromide of potassium, in a recent number of the *New York Medical Record*, by Dr. Carr, he enunciates the prevalent idea thus: "If the brain is already without the proper supply of blood, the extra amount which will be removed from it by the action of the bromide of potassium, will not only not benefit the patient, but will probably increase the force of the paroxysmal seizure." Now, the results of the experiments with this drug on animals carefully collated, as well as those of clinical experience, go far towards demonstrating the fact that the least important of the effects of these agents is their vaso-motor influence; that their most important action is in controlling, and, if pushed far enough, almost obliterating *reflex* actions. First, by a direct effect on the nerve-*centres*, the medulla oblongata and cord especially; secondly, on the *peripheral* nerves, that is, blunting the susceptibility of the latter to painful impressions, and also the receptivity of the cord as to these impressions.* I refer to one corroborative clinical fact. In the case of a lady who had bled to the very verge of death from metorrhagia, who was insensible, and at one time pulseless, but who, upon partial recovery, fell into an alarming condition of nervousness and insomnia, which failed to yield to opium, chloral, etc., I suggested to my friend the late Prof. G. T. Elliot, who was sharing the responsibility with me, to try the bromide of potassium. He at first scouted the idea, but finally agreed, and we were gratified to find that it acted as it ordinarily does in cases supposed to be suffering from hyperæmia.

This important virtue of the bromides is well exhibited in preventing sea-sickness, and the vomiting induced by anæsthetics, and the unpleasant effects of opium and quinine, which clinical experience has recently taught me. But this property has special applicability in the treatment of certain functional diseases of the heart, the history of a case of which, now under treatment, will serve as an illustration.

A. M., a young man of fair constitution, but of a family decidedly addicted to insanity and neurotic affections, applied at my office about four weeks ago, during my absence from home, and was seen by Drs. J. C. Young and G. W. Murdock. He had left his home in the West on account of heart trouble, which had annoyed him for some months; he described it as a fluttering, and a painful consciousness of its action, the impulse being very strong. This was increased by exertion of any kind. He is not addicted to excess of any description; takes neither liquor nor tobacco. He looks pale, but is not

* It has been shown by experiments on animals, that the effect of the bromides applied directly to the nerves is very decided, and that it has therefore an effect on the peripheral nerves independent of that through the nerve-centres.

anæmic; has a good appetite and digestion; sleeps well. Four years ago had what he calls rheumatism, but it was probably myalgia. Complains now of a sort of stiffness across the chest and shoulders anteriorly. Pulse good. They examined him, and found a distinct bruit with the first sound, heard at base and apex; but after conversing with him for a time, and re-examining him, they were surprised at finding no bruit whatever. This happened at two other visits. There was no hypertrophy. On my return, I recognized the case as similar to at least two which I had seen some years ago, and, on making him change his position, the sound would change from base to apex, or sometimes disappear altogether at the same visit. When standing, merely bending well forward would alter the bruit, or cause it almost to disappear. But these changes did not, at different visits, correspond with the same positions; sometimes the sitting posture developing a louder bruit than the standing; sometimes the reverse, sometimes causing its entire disappearance; sometimes a walk, twice across the office, would develop the sound; sometimes have no effect. This is the peculiarity of the cases, and I do not think that such cases are distinctly referred to in any systematic work. Tobacco causes symptoms somewhat similar. The bruit is louder, and yields readily to abstinence from the cause. It is easily distinguishable from the symptoms of anæmia. One case, I remember, was in the person of the coachman of a friend; he was the picture of health, and had no *symptoms*, if I remember rightly, but presented himself for examination. Greatly surprised at finding a bruit, and examining him again and again, and in different positions, the same phenomena as above were developed. Seeing me puzzled about him, he said that he had been in the employ of Dr. Wm. Stokes, of Dublin, and was examined by him. The doctor, he said, called the attention of O'Brien Bellingham, who happened to be in the office, to his case. Cases somewhat similar to these have been observed by other physicians occasionally, especially in connection with *chorea* and other nervous diseases; but the sound has not been described as varying with a mere change of position, or sometimes with no change at all, at the same visit. Although these marked cases are comparatively rare, the same pathological conditions most likely have an existence, and exert an injurious influence in cases of organic disease, for the apprehension excited by a consciousness of this ailment, especially when operating on a nervous temperament, would be apt to induce such an effect on the nerve-centres, and so on the little cardiac ganglia which preside over special sets of fibres of the heart, as to produce the irregular muscular action, or want of unison in action, which is probably the cause of the phenomenon; thus not only modifying the *organic* bruit, but impairing still further the function of the organ.

To return to the history of A. M., I advised *digitalis* as a *test*, not on the supposition that it was the appropriate remedy, also the acid phosphate and the syrup of the phosphates. In a few days he returned and said he was worse, that the thumping was more distinct. He then informed me that he had taken digitalis before, and with the same result. The phenomena on auscultation were not altered. Directed the discontinuance of the remedies, and to take a teaspoonful of cod-liver oil three times a day, and thirty grains of the bromide of potassium three times a day. Four days later he returned, and an examination showed no change. But, on inquiry, it was found that he had neglected to take the bromide. He was now ordered positively to commence this at once. Dec. 3rd, three days later, he presented himself, and neither Dr. Young nor I could detect any bruit, in any position in which we could place him, and on repeated examination. This was the more remarkable, if caused by the medicine, since his mind had been very much disturbed by the sudden death of the mayor of New York, whose symptoms, he imagined, closely resembled his own. Directed the medicine now twice daily. Dec. 5th. Examined the patient to-day with Dr. Young, and although he had been exercising considerably, there was no bruit in any position. There is a want of correspondence, as is often observed in these nervous cases, between the powerful impulse of the heart and the strength of the pulse. Directed to continue the medicine three times a day. We may readily imagine why this drug is so universally applicable in disease, as to draw down, on those who use it largely, the charge of hobbyism, since there is almost no complaint in which, at some stage, there may not be this undue impressibility of the nerve-centres or peripheral nerves, and consequent undue reflex activity, calling for the regulating power of the bromides.

In concluding my remarks on the action of special drugs, I refer to *conium*, which was fully discussed in Dr. Peter's paper, at our last meeting, only to direct attention to another reliable preparation, and the only one for hypodermic medication; also to a valuable paper by Dr. J. Wilkie Burman in the West Riding Asylum Reports on its use in acute mania, in promptly subduing excessive muscular action. The succus conii, so much relied upon at one time in Great Britain, is now pretty much abandoned as uncertain, and inconvenient for its bulk. The fluid alkaloid *conia* has been attempted by their most eminent manufacturers, as Duncan and Flockhart, Morson, etc., but according to Dr. Burman, with uncertain results, until T. and H. Smith, of Duke St., Edinburgh, furnished their preparation, which, he says, he has always found reliable. The others he found not only inefficient as regards expected results, but dangerous in producing unexpected phenomena. The preparation of the celebrated Morck does not correspond in appearance with the description of the Smiths', and Mr. Nurgaard, of this city, has promised to import some of the latter.

As regards the use of these active neurotics, allow me again to advert to the matter of *doses*. It is certain that most of the failure and disappointment, often complained of, has resulted from inefficient doses. We may, I think, give this as a rule of action: that the drug cannot be said to have had a fair trial unless it has relieved the symptoms or produced its toxic effects. With regard to the bromides, strychnia, opium, atropia, physostigma, this is especially the case; and in very dangerous cases, the toxic effects need often to be very pronounced. The doses, in fact, are as various as the temperaments and idiosyncracies of the patients. Every one is aware of what enormous doses of opium are borne, in certain conditions of the system, with impunity. Large doses of some of these drugs have already been adverted to, and I refer to a very interesting case in the last number of the *London Practitioner*, for an account of the successful employment of immense doses of physostigma, the patient a physician, and a man of note. He took, in four days, of the solid extract of a reliable manufacturer, sixteen, forty-eight, fifty-seven, and seventy-two grains. At one time serious paralysis was induced. But the urgency of the disease, tetanus, required a perseverance with the drug.

As many of the topics discussed in this paper are still considered *sub judice;* as the observations, which the author has had the honor to present this evening, are intended rather as suggestive than as demonstrative, and as he is aiming at truth rather than the establishment of theory, he invites your most unrestricted and impartial criticism, and holds himself ready and anxious to receive correction and instruction on these most obscure and difficult problems of medical science.

COLD SPRING, Dec. 6th, 1874.

---

NOTE.

Since this paper was written, other cases, so strikingly illustrative of the correctness of the opinions expressed by the author have fallen under his notice, that he feels justified in adding them in the form of a postscript.

His attention was called to the first case by Dr. Marion Sims. A member of this Society, on going to visit a patient, got his feet and legs wet in a violent rain ; he sat with the patient for a couple of hours, and soon after his return home was attacked with a violent chill, and all the usual symptoms of intermittent fever. He has since continued to have a regular recurrence, notwithstanding appropriate treatment. He has lost a great deal of flesh and strength during the attack. When we take into consideration the fact that, as was understood, he had never previously had the disease, that he was in perfect health at the time, the conclusion seems almost irresistible that the exposure was the most important, if not the only factor in the outbreak. Malaria is so prevalent everywhere, that, although living in a very healthy quarter of the city, he *may* have had some contamination. But it is not at all likely that this, if he had it, would have given any indication of its existence, had he not subjected himself to the existing cause. It is proper to add that the opinion of the author does not coincide with that of Dr. Sims, who holds to the old theory.

The author was to-day applied to, as United States Examining Surgeon, by the Rev. B. F. Bowen, of Cold Spring, for a certificate, in consequence of a wound received in the attack on Petersburg, in 1865. He was a chaplain in the army, but, moved by a patriotic impulse, he joined the ranks, and in a charge, and when stooping forward in the act of firing, he received a wound in the face and the shoulder. He says it felt at first as if a corkscrew were thrust into the part; then he was conscious of a terrible "concussion of the brain." He lay in front of the works from morning until night. He says that, at one time, during the probing of the wound, something like an electric shock passed from it towards the angle of the jaw, thence to the brain, and he became "*blind*" for an instant, but not syncopal or unconscious. Three times since then the same effect was produced by the same cause; when the probe reaches a certain point, at or near the bottom of the wound, or the non-fistulous tract, the electric-like sensation and the transient amaurosis occur. This operation was once done by a brother-in-law, J. W. Robathan, of Scranton; once by Dr. Stone, of Almont, Mich., a former surgeon in the army, and once by Prof. Gunn, then of Detroit. The wound has affected him seriously, both mentally and physically, and it is telling on his nervous system especially, more and more from year to year. Any attempt at manual labor affects his left arm and his brain. Sometimes, when he is preaching or lecturing, his mind, for an instant, or a few moments, is a blank, and his memory gone. The same occurs when reading or thinking. He has other singular sensations referable to the region of the heart, which are, at times, still more alarming. These almost always occur while in the recumbent posture, and just as he is getting to sleep. A sensation like a powerful electric shock passes through the heart, and he leaps out of bed in a sort of fright and bewilderment. When in a strange house, it disturbs him so that he cannot sleep the remainder of the night. During a thunder-storm, the lightning seems always to go through his heart. At times the sensation, when lying down, is not so severe, and is like a vacuity or "goneness." There are almost constant twinges, and some soreness on the injured side of the neck, shooting up *superficially* towards the scalp, but entirely different from the alarming sensations above described, and which always appear to go deeply into the brain. Mr. Bowen's general appearance is that of fair health. He is a thoroughly educated, and a very intelligent and energetic man, and a man of great fortitude.

The ball entered the left side of the cheek just in front of the line of the masseter muscle, passed inside of the lower jaw, without injuring it, downwards behind the sterno-mastoid and the jugular vein, avoiding the carotid and other large vessels, very superficially towards the outer third of the clavicle, then between the scapula and ribs, to about the head of the lower third of the former, where it lodged. Six months after the wound was received, a soreness was observed at a point an inch or more above the junction of the outer and middle third of the clavicle, and an opening soon formed, from which two fragments of bone, from a half to three-quarters of an inch in length, were discharged. An opening still exists here, and discharges bloody pus, and sometimes, after exertion, blood.

It has been a question, the patient says, among medical men, whether the necrosed bone, which probably causes the symptoms, is the scapula or the rib. There is, I think, good reason to suppose that it is the latter, and it will now be a matter for consultation whether an explorative operation ought not to be performed with a view to ascertain, and, if possible, remove the source of irritation of the nervous system, whether it be the bone or a bullet.

I have not the space at command to comment on the many interesting features of this case, as bearing on what has gone before; especially on the cases of amaurosis previously published by the author, to which I add the experience of M. Trousseau (Clinique Med. de l'Hotel Dieu de Paris), who speaks of temporary amaurosis caused by the tickling of worms in the intestines of children, a very common nervous symptom of their presence being also convulsions.

S. Weir Mitchell* records two cases similar in some respects to that here related. In one, the ball entered behind the ramus of the jaw, in front of the sterno-mastoid, and traversed the neck to the other side. Although there was no direct injury to the brain whatever, the man fell and remained unconscious for about half an hour. He then got up and marched off to join his regiment. I cannot here even epitomize the very interesting history of this case as given by Dr. Mitchell. He ends by saying: "In the case of Mooney, the symptoms consisted in loss of memory, vertigo, cephalalgia, and presenting such a degree of gravity as to necessitate his entrance into hospital long after the cure of the wound." Symptoms not very different from those of Mr. Bowen.

* Des Lesions des Nerfs. Fr. Ed. Paris, 1874. Observation 58[e].

The other was the case of Captain, afterwards Commodore Stemble. Here the ball also took a course corresponding somewhat to one part of the track of the ball in Mr. Bowen's case. Entering just above the os hyoides, it passed backward and downward, on the right side, under the sterno-mastoid, and came out near the superior angle of the scapula. Here there was temporary loss of vision in the right eye, slight ptosis remaining after two years, contraction of pupil, deficiency of sweat on right side, and exaggeration of this secretion on the left side of face and neck.

Dr. Mitchell remarks that there seems to have been some disorder of the great sympathetic in this case; but that he is inclined to consider the symptoms as due to reflex action. Such is probably the explanation of Mr. Bowen's symptoms.

